# Time-Varying Wing-Twist Improves Aerodynamic Efficiency of Forward Flight in Butterflies

**DOI:** 10.1371/journal.pone.0053060

**Published:** 2013-01-16

**Authors:** Lingxiao Zheng, Tyson L. Hedrick, Rajat Mittal

**Affiliations:** 1 Department of Mechanical Engineering, the Johns Hopkins University, Baltimore, Maryland, United States of America; 2 Department of Biology, University of North Carolina at Chapel Hill, Chapel Hill, North Carolina, United States of America; University of Zurich, Switzerland

## Abstract

Insect wings can undergo significant chordwise (camber) as well as spanwise (twist) deformation during flapping flight but the effect of these deformations is not well understood. The shape and size of butterfly wings leads to particularly large wing deformations, making them an ideal test case for investigation of these effects. Here we use computational models derived from experiments on free-flying butterflies to understand the effect of time-varying twist and camber on the aerodynamic performance of these insects. High-speed videogrammetry is used to capture the wing kinematics, including deformation, of a Painted Lady butterfly (*Vanessa cardui)* in untethered, forward flight. These experimental results are then analyzed computationally using a high-fidelity, three-dimensional, unsteady Navier-Stokes flow solver. For comparison to this case, a set of non-deforming, flat-plate wing (FPW) models of wing motion are synthesized and subjected to the same analysis along with a wing model that matches the time-varying wing-twist observed for the butterfly, but has no deformation in camber. The simulations show that the observed butterfly wing (OBW) outperforms all the flat-plate wings in terms of usable force production as well as the ratio of lift to power by at least 29% and 46%, respectively. This increase in efficiency of lift production is at least three-fold greater than reported for other insects. Interestingly, we also find that the twist-only-wing (TOW) model recovers much of the performance of the OBW, demonstrating that wing-twist, and not camber is key to forward flight in these insects. The implications of this on the design of flapping wing micro-aerial vehicles are discussed.

## Introduction

Insect wings deform to varying degrees during flapping flight, and the kinematics of wing deformation [1]–[3] as well the underlying structural features [4], [5] have been investigated in a variety of species. Chordwise deformation resulting in a time-varying camber is found in most insects including hoverflies [6], bumblebees [7] and moths [8], [9]. Spanwise deformation in the form of wing-twist has also been reported in large insects such as locusts [10]. Butterflies, which have some of the largest wings among insects, exhibit noticeable chordwise and spanwise deformation [3]. Despite many efforts to characterize the deformation modes of insect wings and their relation to the inertial and aeroelastic loads experienced by the wings during flight, studies directly quantifying both the deformation and its effect on the aerodynamic forces produced by the wings, remain rare.

Since the inclusion of wing deformation increases the complexity of the analysis, most studies of the aerodynamics of insect flight assume the wings to be rigid and flat (e.g. [11], [12]). Some studies have introduced flexibility by varying the flexural stiffness of a flat plate [13], [14] while others used deformable wing models with simplified structural elements [15], [16]. These approaches help quantify the aerodynamic effects of deformation, but may not reproduce the types of deformation exhibited by insect wings. To more directly address the effects of camber and twist deformation, Du and Sun [17] constructed a flexible-wing model for hoverflies (*Eristalis tenax*, Linnaeus), assuming an approximately linear spanwise wing-twist and a wing camber that was uniform across the span and constant during each half-stroke. Their model showed that camber is more important than wing-twist in determining the aerodynamic performance in the hovering flight of hoverflies. Nakata and Liu [18] performed an analysis of the aerodynamic performance of a hovering hawkmoth using a computational model that included the effect of flow-structure interaction on the wings, and they found that the lift-to-power ratio of rigid wings was about 9% lower than that of the flexible wings. The above studies therefore indicate that for hovering flight, the advantage of wing flexibility is relatively modest, and furthermore in most cases, attributed to deformation in camber.

Unlike hovering flight where the generation of lift is the key determinant of wing motion, in forward flight, the wings also have to generate a net positive thrust during each flapping cycle to maintain the forward velocity of the animal. This has to be accomplished by the wings by generating positive forward force (thrust) in some phases of the cycle and reducing the negative force (drag) on the wings in other phases. It is therefore not clear if the observations regarding the effects of wing deformation on hovering flight extend to insects in forward flight. An attempt to understand the effect of wing deformation during forward flight was made by Walker *et al.* [10] who characterized the wing deformation of a tethered locust *Schistocerca gregaria* in a wind tunnel using stereo videogrammetry. Young et al. [19] conducted computational fluid dynamic (CFD) simulations of this configuration and derived one wing model with no camber and another with neither camber nor twist. Their results indicated that flat-plate wings were about 15% less efficient (in terms of total aerodynamic force to power ratio) and that twist and camber deformation contributed almost equally to this total deficit. The study therefore confirmed that the role of wing deformation might be dependent on the flight condition.

In the current study we explore this issue further by studying the effect of time-varying twist and camber on the free forward flight of the Painted Lady butterfly *Vanessa cardui*. These butterflies are large relative to most volant insects and have broad wings with the forewing and hindwing functioning as a single surface, leading to wing aspect-ratios (see Table 1 for a comparison of the mass, wing length, wing area, aspect-ratio and wing loading of this butterfly with other insects) that are lower than the insects previously studied [10], [17], [19]. This lower aspect-ratio potentially enhances the effects of camber relative to those found in species with high aspect-ratio wings. The size and shape of the wings lead to larger deformations than observed in most other insects, making butterflies excellent model organisms for examining the effects of wing deformation. Furthermore, by using freely-flying animals, the current study avoids any spurious effects that might be associated with tethered insect flight [20], [21].

**Table 1:** Comparison of mass (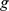), wing length (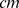), total wing area (), aspect ratio and wing loading (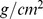) for different insects.

Insect	Mass (*g*)	Wing length (R) (*cm*)	Wing area (S) (*cm* ^2^)	Aspect ratio (4*R* ^2^/*S*)	Wing loading (*g/ cm* ^2^)
Vanessa cardui	0.29	3.01	11.4	3.18	0.0254
Schistocerca gregaria [35]	2.08	5.33	29.9	3.80	0.0696
Manduca sexta [36]	1.41	4.96	17.4	5.66	0.0812
Drosophila melanogaster [37]	0.001	0.239	0.0382	5.98	0.0262

**Figure 1:**
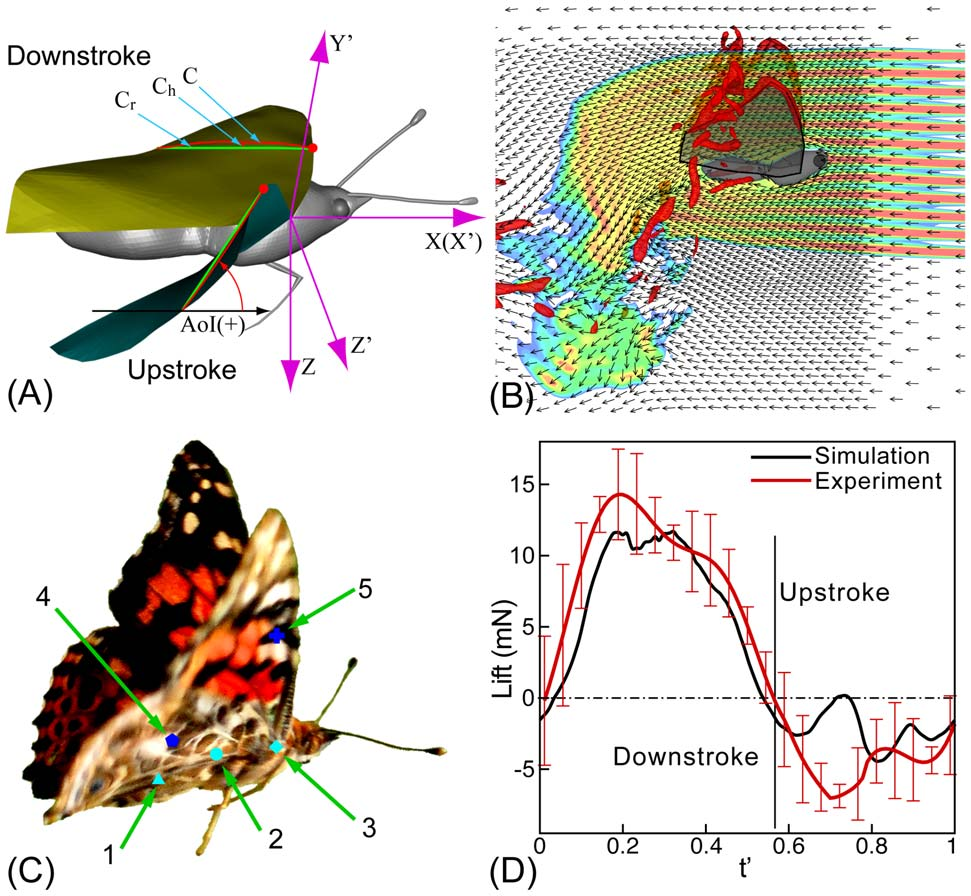
Key variables and validation results. (A) Schematic defining the variables used to parameterize the kinematics and deformation of the wing in the current study. (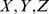) and (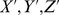) are lab and wing-attached coordinated frames respectively. The wing shape and position at the middle of downstroke (gold) and upstroke (aqua) are shown. The red dot identifies the leading-edge and 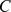 is the cambered shape associated with the wing surface at one spanwise location in both wing positions. The local camber is defined as the ratio of maximum camber (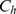 in the figure) to the local chord length (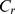, which is the straight line joining the leading and trailing edge of 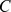). The angle-of-incidence (AoI) is defined as the angle between 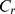 and the 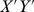 plane. (B) Simulation result for the observed butterfly wing (OBW) in forward flight showing streaks mimicking smoke traces and vortical structures at early downstroke. (C) *Vanessa cardui* with its brightly colored wings. Center-of-masses (CoMs) for different parts of the butterfly are marked. Point 1: abdomen; Point 2: whole body; Point 3: head and thorax; Point 4: hind wing and Point 5: fore wing. (D) Comparison of the lift force predicted by the simulation and the experimentally estimated value. The error-bars indicate the uncertainties in the experimental estimate.

**Figure 2:**
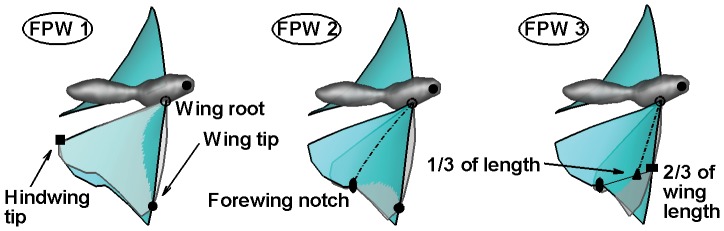
Description of key features in the generation of flat-plate wing (FPW) models. Each FPW model is aligned to a unique set of three selected points on the OBW as shown: wing root, wing tip and hindwing tip for FPW 1; wing root, wing tip and forewing notch for FPW 2; wing root, point at the leading-edge at 2/3 wing-span, and 1/3 wing-chord at this spanwise location for FPW 3.

The study employs a Navier-Stokes (NS) based computational model of the flight aerodynamics which are based on a precise reconstruction of the observed twist and camber of the butterfly wings (Fig. 1A). We then compare these results to simulations for a set of synthetic model wings (Fig. 2), each of which incorporates specific features of the motion and deformation of the actual wings, thereby revealing the effect of time-varying deformation on flow features, force production and power expenditure. We find that the effect of deformation observed here is more than three-fold larger than that reported by Young et al. [19] for a tethered locust and roughly five times larger than that reported by Nakata and Liu [18] for a hovering hawkmoth. Furthermore, despite the morphological factors associated with butterfly wings that appear to favor camber, we find that wing twist is the dominant wing-deformation feature in forward flight of the butterfly, improving lift production by upwards of 29% and lift-to-power ratio by upwards of 46% over flat-plate wings. In contrast, camber is found to provide only minimal (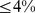) improvement in these quantities.

## Materials and Methods

### Experimental Setup and Procedures

The Painted Lady butterfly (*Vanessa cardui*), well known for its orange wings with striking black and white spots (see Fig. 1C), was used for this study. The body of a butterfly specimen was laser-scanned with 0.05 inch resolution and a 3D geometrical model created from this scan. Photographs of the wings were used to generate zero-thickness membrane models of the wing wherein each model was made up of 6232 triangular elements. The flight and wing kinematics of butterflies were quantified using a high-speed videogrammetry setup composed of three synchronized Redlake Y4L high-speed cameras paired with Nikon AF Nikkor 24–85 mm f/2.8–4D IF close-focusing lenses. Given the wing flapping frequency of approximately 20*H z*, the cameras were operated at 2000*H z* record rate with a shutter duration of 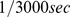 and a 1024 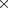 512 pixel resolution.

We collected approximately 100 separate recording of the butterflies flying freely inside a 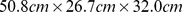 glass chamber with the cameras positioned outside the chamber. These videos were then examined carefully and we selected from these one particular recording where the butterfly was flying straight and level at near constant velocity and for which the image acquisition from all three cameras was of high quality. Taking advantage of natural markings as well as other easily identifiable locations such as the wing root or tip we reconstructed the 3D deforming wing kinematics at 9 instants during a flapping cycle by measuring the 3D location of 35 points on the wing surface from this recording. Three-dimensional coordinates of these points were computed using direct linear transformation [22]; the average 95% confidence interval for the photogrammetric reconstruction of each point was 0.19 mm. These 35 points were then used to update the locations of all 3118 vertex points in the triangular-element wing mesh via linear interpolation. The repositioned wing surface mesh was then smoothed in the following way: the coordinates of the measured points and those at wing edge were held unchanged while the others were replaced by the average value of surrounding nodes. Once the smoothed mesh was obtained for the complete flapping cycle, we used a cubic spline to interpolate the surface mesh in time to produce a high temporal frequency (900 time-steps per flapping cycle) input for our CFD solver.

### Wing and Body Kinematics

The wing length, mean chord and flapping frequency of the particular butterfly examined in this work are 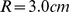, 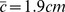 and 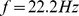 respectively. The wingbeat amplitude 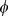 (defined as the angle between the lines joining the wing root to the wing tip at the top and bottom of the stroke) is 120°, the stroke-plane angle 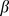, defined as the angle between 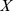-axis and the projection onto the 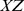-plane of the line joining the wing-tip positions at the top and bottom of the stroke, is 78.5°. The average wing-tip velocity during one cycle is 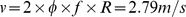. The Reynolds number 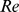 for the butterfly in forward flight is 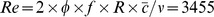. We assumed a constant forward velocity of 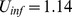 m/s which is the average velocity recorded for the butterfly during one wingbeat. The body pitch (angle between the body and horizontal plane) was set to a constant value of 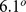 in accordance with our measurements. In actuality, the forward velocity varies slightly during the flapping cycle (the the standard derivation in the forward velocity is about 4% the average value). The standard deviation in body pitch angle is about 29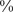 of the corresponding average value. However, this larger cyclical variation in the pitch angle is expected to have a minimal effect on the computed results firstly because the kinematics of the wings in our model are derived from measurements of wing motion which intrinsically incorporate any body-pitch-induced variations. Second, the aerodynamic force produced by the body, which might be affected by cyclical variations in the body pitch, is less than 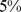 of the wing-induced force and therefore of little dynamical significance. Finally, the emphasis of the current study is on lift generation and body produces no measurable lift.

**Figure 3:**
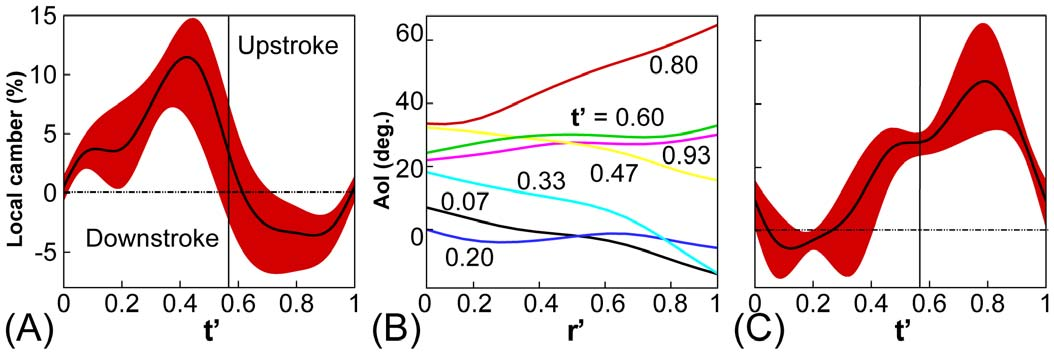
Local camber and twist of the butterfly wing. (A) Time variation of local maximum camber normalized by local chord length 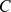, through the wingbeat at different span-wise position. Note that 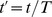, where 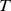 is time period for one wingbeat. A positive value means the leading and trailing edges of the wing bends downward and vice verse. (B) variation of local AoI across the wing (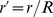, where 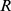 is the wing length) which is a measure of wing twist. (C) variation of local AoI through the wingbeat. In Fig. (A) and (C), the red shaded region shows the spanwise variation of the AoI and the black line denotes the mean value.

Figure 1A defines a local angle-of-incidence (AoI) and local camber, which are used to quantify the chord-wise (camber) and spanwise (twist) deformations of the deforming butterfly wing (OBW) model, with respect to the wing-attached frame of reference (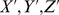). Figure 3A shows the local camber through the flapping cycle at different spanwise positions. Temporal and spatial variations in AoI through the wingbeat cycle and along the spanwise axis of the wing are shown in figures 3B and 3C, respectively.

### Navier-Stokes (NS) Flow Modeling and Quantitative Validation

The governing equations used in current study are the three-dimensional unsteady, viscous incompressible Navier-Stokes equations:
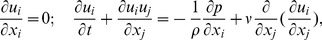
(1)where 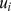 are the velocity components, 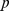 is pressure, and 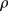 and 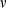 are the fluid density and viscosity, respectively. A cell-centered, collocated (non-staggered) arrangement of the primitive variables (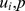) are used to discretize the above equation. The face-center velocities, which satisfy mass conservation, are also calculated in addition to the cell-center velocities (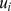). A multi-dimensional ghost-cell methodology, which falls in the category of sharp-interface discrete forcing immersed boundary methods [23], is used to incorporate the effect of the immersed boundary on the flow. As noted earlier, an unstructured grid with triangular elements is employed to represent the surface of three-dimensional bodies such as the insect wings and body which are immersed into the Cartesian volume grid. The flow solver employs a second-order accurate fractional-step scheme for temporal discretization and a central-difference based spatial discretization that is also locally and globally second order accurate. Further details regarding the current method as well as validation and verification studies can be found in Refs. [23], [24] and [25].

In the current study, the baseline simulation has been carried out on a non-uniform 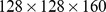 point Cartesian grid and 900 time-steps per flapping cycle. The overall computation domain has a size of 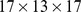 in terms of the wing span and a cuboidal area around the butterfly of size 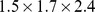 win-spans has a high-resolution uniform grid which is designed to resolve the near-wake vortex structures. Grid independence is assessed by simulating the same case on two other grids: one with 70% more and the other with 50% fewer grid points than the baseline grid. The difference of the root-mean-square (RMS) lift from the baseline case is 4.9% for the coarse grid and 0.2% for the finer grid. The corresponding values for RMS drag are 1.3% and 0.88% respectively. These results indicate a high level of accuracy in the aerodynamic forces computed on the baseline grid, as well as the flow features that produce these forces.

Figure 1b shows simulated “smoke” streak visualization of the computed flow during the early downstroke of the OBW model. The flow that has passed over and between the two wings before the start of the downstroke is deflected downwards and forms a large vortical region below and downstream of the butterfly. This, despite the differences between the two configurations, is qualitatively similar to the smoke visualization experiments of a Peacock butterfly in tethered flight (see [26]).

For quantitative validation, we have compared the time-varying lift computed by the NS simulations against estimates of the same quantity from our experiment (see Fig. 1D). Lift estimates were obtained from the experiment by tracking the center-of-mass (CoM) of the butterfly in space and time using stereo videography and DLT. The overall CoM of the body (shown as point 2 in Fig. 1C) at any time-instant was estimated by separately tracking the CoMs of the abdomen (point-1), head and thorax (point-3), and the two hind (points 4) and fore wings (points 5). The CoMs of the abdomen and head-thorax segments were found by assuming a constant body density and determining the volume centroid of the segments. The CoM of the hind and fore wings was found by using a plumb-line since the wings do not have a uniform density; more details on these procedures are available in [27]. The vertical coordinate of the CoM was low-pass filtered in time to remove noise and the net vertical acceleration of the body estimated by computing the second derivative with respect to time of the vertical coordinate and adding a gravitational acceleration of 9.81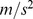 to the result. This estimate is subject to uncertainty originating firstly from the videographic resolution of the various components of the body. Secondly, the fractional mass of the head, thorax and abdomen also affects the overall CoM calculation and can vary from individual to individual as well through time due to feeding history. However, we only employed average values for these quantities which were recorded for a set of similar specimens that were sacrificed and their body parts weighted separately [27]. The variation in these values over the set of specimens also provides input for estimation of experimental uncertainty.

Figure 1D shows a comparison of the lift force produced in the simulation and the experimental estimate. The experimental uncertainties described above have also been estimated and are included in the plot. It is noted that during downstroke, the wings produce a large and extended lift peak, while during upstroke, the wings produce a negative lift with a magnitude that is much smaller than that during downstroke. This temporal variation is matched fairly well in the simulation with the highest deviation occurring during early upstroke. We note that even in this carefully selected video, the wing kinematics of the butterfly are not perfectly periodic, but the CFD simulation assumes periodic motion from flap to flap. The differences between periodic flapping kinematics for the CFD and the actual flapping kinematics are greatest at the end of downstroke and this produces the discrepancy between the experimental and CFD based lift at this phase. However, in spite of this discrepancy, the Pearson correlation between the measured and predicted lift profiles is 0.94 which indicates a good match between the two. Furthermore, the root-mean-square lift computed from the simulation is 6.7 mN and the corresponding value from the experiment ranges from 6.2 to 10.2 mN. Finally, the peak lift during downstroke, which is particularly essential for keeping the animal aloft is predicted to be 11.7 mN in the simulation and this value lies within the experimental estimate of 11.5–18.0 mN. All of these comparisons provide an objective assessment of the fidelity of the computational fluid dynamics model.

### Synthesis of Flat-Plate (FPW) and Spanwise Twist-Only Wing (TOW) Models

FPW models are constructed by fitting a plane through a set of three points on the butterfly wings during the flapping cycle. In order to capture a wide variation of FPW configurations and kinematics, we have synthesized three distinct FPW wing models by tracking different point-triples for each model. The wing configurations for the observed butterfly wing (OBW) as well as the corresponding flat-plate wings are shown in Fig. 2; FPW 1 is based on fitting a flat plate to the combined fore and hind wings whereas FPW 2 and FPW 3 attempt to match the entire forewing and the forewing leading-edge respectively. In order to isolate the effects of span-wise deformation (twist) from chord-wise (camber) deformation, we have also constructed a twist-only-wing (TOW) model where the local cambered crossection of the OBW wing (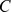 in Fig. 1A) is replaced with a straight chord that has the same effective AoI (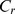 in Fig. 1A). Thus, unlike the FPW wings which have neither camber nor twist, the TOW wing has no camber but a local AoI (and therefore twist) that matches the corresponding AoI for the OBW model.

## Results

### Wing Deformation and Kinematics

The observed butterfly wing (OBW) with time-varying deformations produced forces similar in magnitude and timing to those measured from the actual animal (Fig. 1D). The computed flow structure also exhibits commonly observed features such as a large leading edge vortex (LEV) and a simulated smoke trail visualization (see Fig. 1B) produces flow features similar to those reported for a tethered Peacock butterfly [26].

As expected, this the OBW model exhibits substantial time-varying deformation in camber (Fig. 3A) as well as twist (Fig. 3B), with camber as a fraction of chord length reaching 15% during downstroke and 7% during upstroke. In contrast to camber, the wing exhibits large (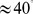) twist during both phases of the flapping cycle. (Fig. 3B). The angle-of-incidence (AoI) also shows significant variation in time (Fig. 3C); during downstroke, the wing maintains a small magnitude implying a mostly horizontal wing orientation, whereas a large (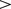 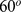) AoI is noted during upstroke. Also notable is the large spanwise variation in the local AoI. These variations, which reach up to about 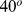, are a consequence of the combined effects of spanwise camber variation and wing twist.

**Figure 4:**
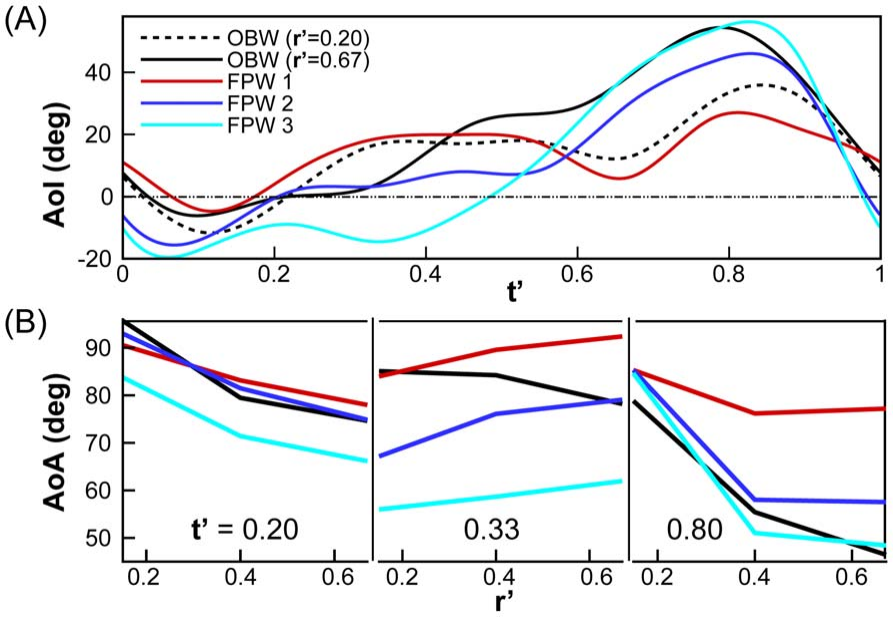
Time-variation of AoI and spanwise variation of AoA. (A) Time-variation of the angle-of-incidence (AoI). Note that the AoI of the TOW along the span exactly matches that of the OBW since they have identical wing twist. (B) Spanwise variation of the angle-of-attack (AoA) during mid-downstroke and mid-upstroke for different models sampled at three locations along the wing.

The kinematic features of the flat-plate wing models (see Materials and Methods) are compared to the OBW model in Fig. 4. Figure 4A shows the AoI during one wingbeat for the FPW models; for the OBW and TOW wings, we plot the AoI at wing root (20% span) as well as the 67% span where these wings produce maximum lift per unit area. Note that the AoI of the FPW 1 wing is close to the AoI near the wing root of the OBW model since we match the motion of wing root and hind-wing tip for these two cases (see Fig. 2). On the other hand, FPW 2 matches the AoI of the deformable wing at 67% span during downstroke while FPW 3 matches the AoI at this location during upstroke. As shown in Fig. 4A, the three FPWs span a large range of AoI variation indicating that between them, they cover an extensive repertoire of possible wing strokes with a flat wing.

The flow over the wing is driven primarily by the sectional angle-of-attack (AoA) and estimates of this quantity can be obtained as AoA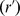 = AoI
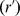
+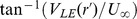 where 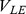 is the vertical velocity of the wing leading-edge. Since most of the lift is generated by the distal portion of the wings, we compare the AoA at 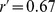 for the wing models at mid downstroke (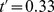) and mid upstroke (at 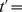 0.80) in Fig. 4B. There are significant differences in AoA between the various wing models; first during mid downstroke, where for the OBW wing model (and the TOW, which has the same AoA as the OBW) AoA decreases with 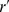 due to wing twist whereas the FPW wing models all show increasing AoA with 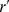. During mid upstroke, the AoA variation with 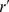 of the OBW is similar to that of FPW 2 and FPW 3 but FPW 1 shows a significantly higher magnitude of AoA.

### Instantaneous Lift, Thrust and Power

**Figure 5:**
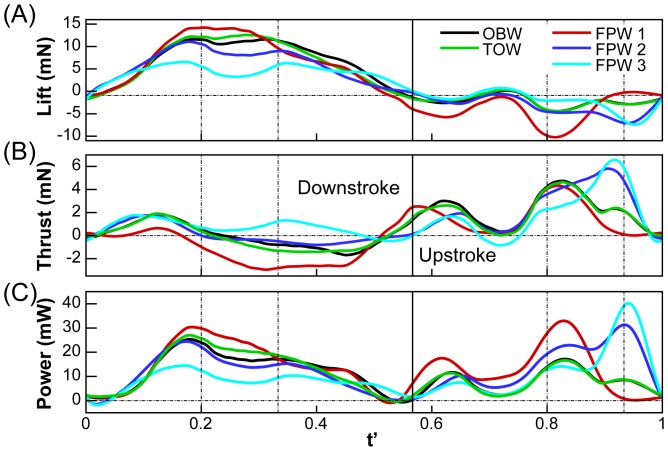
Comparison of instantaneous (A) lift, (B) thrust and (C) power during one flapping cycle for different models.

The instantaneous lift, thrust, and power (In the current study, the power was calculated as 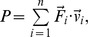 where 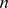 is total number of triangular element on the wings, 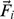 is the aerodynamic forces on each element and 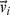 is the corresponding velocity of the element.) obtained from numerical simulations for different models are shown in Fig. 5. Figure 5A indicates that for all the models, a large positive lift plateau is generated during mid-downstroke. FPW 1 produces the highest instantaneous lift during downstroke that is approximately 20% higher than the OBW model. However, the lift-plateau produced by the OBW model is wider, extending from about 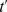 0.17 to 0.33. In fact, the OBW model generates a lift plateau during downstroke that is wider than all the other models except FPW 3. However, the peak value of lift for FPW 3 is only about half that of the OBW model. The TOW model produces a lift-plateau that is marginally higher but somewhat narrower than the OBW model. During upstroke, all the models produce negative lift although FPW 1 exceeds all other models in terms of the magnitude of this quantity. FPW 2 and FPW 3 follow similar trends and are comparable to the OBW wing except at the end of upstroke where they produce greater negative lift. The lift for the TOW model is virtually indistinguishable from the OBW model in this phase of the flapping cycle.

The time-variation of thrust is generally opposite to that of lift. The upstroke produces most of the positive thrust whereas the downstroke produces either little or negative net thrust. For the OBW model, positive thrust during early downstroke nearly balances out the negative thrust during late downstroke. Two clear peaks in thrust are produced for this case during upstroke; one during early upstroke and one during mid upstroke. The FPW 1 model produces similar thrust peaks during upstroke but produces substantially more negative thrust during downstroke. The FPW 2 and 3 wing models produce more positive thrust during downstroke. During upstroke, the second peak in thrust is also noticeably higher than all other cases and occurs later in the stroke; this is primarily due to the rapid rotation of the proximal part of the wings. As with the lift, the TOW model generates a thrust that is similar to the OBW model.

Figure 5C shows the total power imparted to the air by the wings and we find that for all cases, the time-variation of power during downstroke and upstroke follows trends similar to the corresponding variation of lift and thrust respectively. While the power consumed for the OBW during downstroke lies between the FPW 1 wing and the FPW 2 and 3 wing models, the flat plate wing models require more power during upstroke than the OBW wing. The TOW wing shows a power variation during upstroke that is similar to the OBW wing.

### Comparison of Aerodynamic Performance

We examine the overall performance of each of these different wings, the actual observed butterfly wing model (OBW), the TOW and the FPW models 1–3 by comparing the stroke-averaged lift (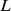), power (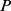), lift-to-power ratio (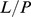), total force (vector sum of lift plus thrust; 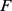) and total force-to-power ratio (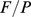) of each of these wings in Table 2. Based on all of these quantities, our simulations indicate that the OBW outperforms all of the other models. However, the comparison also shows that the TOW model performs nearly as well as the OBW, producing a lift and total force that is only 3% and 4% lower respectively, than the corresponding values for the OBW model. The power (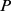) required by the TOW model to produce usable force is also comparable to the OBW with 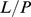 and 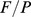 only marginally (about 6%) lower than the OBW model. In contrast, all of FPW wing models substantially under-perform the OBW model; lift production for all the three FPW models lags the OBW model by at least 29% and the total force production by at least 28%. This reduction in usable force is also accompanied by a higher power expenditure leading to an 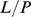 that is at least 46% lower than the OBW model, and a 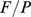 which is at least 30% lower.

**Table 2:** Comparison of mean lift (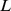), total force(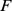), power 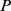, 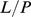 and 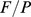 during one flapping cycle obtained from our simulations.

Model	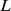 (*mN*)	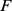 (*mN*)	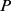 (*mW*)	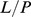 (*N/W*)	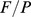 (*N/W*)
OBW	2.93	3.15	10.5	0.279	0.300
TOW	2.85(−3%)	3.02(−4%)	10.7(2%)	0.266(−4%)	0.282(−6%)
FPW1	2.07(−29%)	2.07(−34%)	13.7(31%)	0.151(−46%)	0.151(−50%)
FPW2	1.81(−38%)	2.26(−28%)	12.4(18%)	0.145(−48%)	0.182(−39%)
FPW3	1.42(−52%)	2.06(−34%)	9.81(−7%)	0.144(−48%)	0.210(−30%)

The values in the parentheses are the relative differences from the results of OBW model.

## Discussion

### Wing Deformation in Free Flight

As remarked on from earlier studies of flapping flight in butterflies [3] and as expected from their large wings, the butterfly examined here exhibited substantial wing deformation in twist and camber. The maximum camber reported here, 15%, is slightly greater than that reported previously for hoverflies [10] and locusts [6], but similar to that reported for two other Lepidoptera species [8], [9]. The observed camber is also opposite of that expected for a flat plate with uniform stiffness actuated at the leading edge; such a foil would deform passively in a way that the trailing-edge deflects upward (i.e. negative camber) during downstroke and downward (i.e. positive camber) during upstroke (see for example [28]). Instead, the trailing-edge of the butterfly wing bends downward (i.e. positive camber) during downstroke and upwards (i.e. negative camber) during upstroke; similar wing deformation has been observed for many other species (as in hoverflies [6], bumblebees [7] locusts [10] and moths [8], [9]) and may indicate that the butterfly wing configuration during flight is a result not only of passive deformation due to flow and inertia, but also active wing “morphing”.

The maximum twist in the butterfly’s wing of nearly 40 degrees exceeds the previously reported maximum twist angles of approximately 20 degrees in locust, hoverfly and hawkmoth flapping wings. Furthermore, the timing of maximum wing twist differs among these species. Maximum twist in the butterfly occurred at 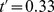 which is mid-downstroke, as well as 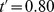 which is mid upstroke. Maximum twist in hawkmoths [29] and hoverflies [2] is reported to occur during the stroke reversal at the downstroke to upstroke transition, and maximum twist in the locust occurred during mid-upstroke. The mid-stroke timing of wing deformation in the butterfly examined here suggests that the deformation is not due to wing inertia, since the acceleration of the wing is small at mid-downstroke (and mid-upstroke), but instead due to aeroelastic effects since the aerodynamic forces are largest at the midpoints of the downstroke and upstroke. The temporal coincidence of maximal wing twist and maximal aerodynamic force underlies the importance of twist to wing performance as revealed by the comparison of OBW and FPW models. Additionally, unlike camber, the observed wing-twist is consistent with that expected from passive mechanisms such as inertia or flow induced torques coupled with the torsional rigidity of the wing.

### Vorticity and Pressure

**Figure 6:**
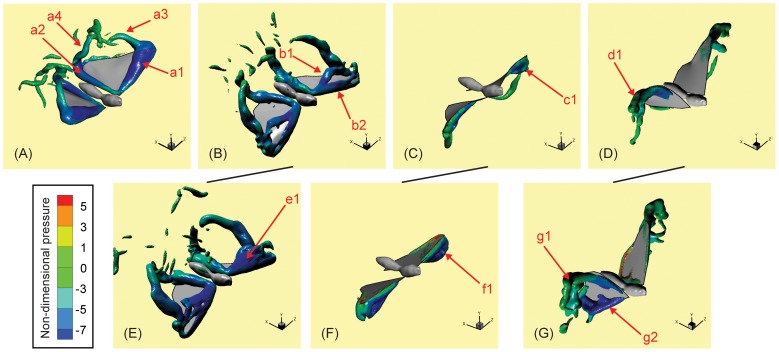
Vortex structures and associated pressure for various cases at selected time-instances. (A–D) OBW model at 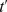 = 0.20, 0.33, 0.80 and 0.93 respectively. (E–F) FPW 1 model at 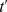 = 0.33 and 0.80 respectively. (G) FPW 3 model at 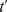 = 0.93. For all the cases the vortices are visualized by plotting one isosurface of the swirl strength (corresponding to a non-dimensional value of 20.5) and pressure is visualized by plotting color contours on the isosurfaces. Salient vortical features are labeled in each panel for ease of discussion.

While all the wing models explored here have the same shape, flapping frequency and amplitude, the presence/absence of time-varying camber and twist generates differences in sectional AoI and AoA (Fig. 4A,B), which in turn induce differences in flow, vorticity and aerodynamic forces. Figure 6 shows three-dimensional vortex structures (colored by magnitude of pressure) at 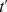 = 0.20, 0.33, 0.80 and 0.93 for the OBW model. The vortex structures are visualized by plotting an isosurface of the swirl strength [30]. At 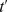 = 0.20, which is early downstroke, we observe the formation of a large leading-edge vortex (LEV) that spans the entire leading-edge of the wing (a1 in Fig. 6A). This vortex is observed to increase in size across the span; it produces a low pressure on the dorsal wing surface and provides much of the lift during early downstroke. Similar LEVs have been observed in other flying insects [11], [12], [19] and butterflies [26], [31]. A smaller attached vortex (a2) is also observed at the proximal edge and posterior tip of the wing, which are in fact associated with the hind wing. This low pressure region also contributes to the total lift and has not been described in other insects. In addition to these two attached vortices, there are also detached vortices; one extending from the spanwise tip of the forewing (a3) and one from the posterior tip of the hindwing (a4). At 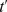 = 0.33 shown in Fig. 6B, which corresponds the period just after mid-downstroke, the original LEV (b1) is close to separating from the wing but a new LEV (b2) is observed to form at the leading-edge. These dual leading-edge vortices have also been reported by other groups using different experimental methods [31]–[33]. The significant positive camber in the distal section of the wing orients the suction pressure associated with the two LEVs in the vertical direction and helps the OBW to maintain high lift.

Figure 6C shows the vortex structures at approximately mid upstroke (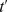 = 0.80); the primary feature is a leading-edge vortex on the ventral side of the wing (c1) which is smaller in size and strength than the LEV generated during downstroke. This difference is a direct consequence of the lower effective angle-of-attack during upstroke (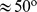; see Fig. 4B) as compared to the downstroke (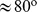) of the distal half of the wing where the LEV is located. While the AoA determines the local aerodynamics of the wing, the partition of force into thrust and lift components is determined by the AoI and we note that the distal portion of the wing achieves its highest AoI (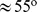; see Fig. 4A) at around 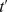 = 0.80. Consequently, the suction pressure on the ventral surface of the wing is directed primarily in the forward direction leading to a high thrust and low lift. We also note that the significant wing-twist at this phase enables the distal portion of the wing to retain a high AoI and in doing so, plays an important role in generating thrust. Figure 6D shows the vortex structures at 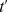 = 0.93 which is near end-upstroke; the primary feature is a tip-vortex (d1) that extends from the ventral surface of the wing-tip out into the spanwise direction. This vortex also produces low pressure on the ventral surface of the wing-tip resulting in positive thrust and a slightly negative lift (see Fig. 5).

FPW 1 produces vortex structures during early downstroke that are similar to the OBW wing except around 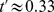 where the slightly higher AoA (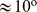; Fig. 4B) of this wing results in earlier separation of the LEV (see e1 in Fig. 6E) and consequently, a sharper drop in lift. The primary difference between the OBW and FPW 1 models is during mid upstroke (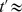 0.80) where an approximately 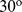 higher AoA for the FPW 1 model (Fig.4B) results in a larger separation (see Fig. 6F ) on the ventral surface and consequently, to significant negative lift. For the FPW 2 and 3 models, the significantly lower lift during downstroke is associated with the lower effective angle-of-attack of the wing (see Fig. 4B) for these models. This results from matching the leading-edge portion of the wing (see Fig. 2 B and C) and it induces a weaker LEV at mid-downstroke. The most significant difference between these two models and the others is during late upstroke; Fig. 6G shows the vortex structures for FPW 3 at 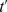 = 0.93 and we note that in addition to a somewhat stronger tip-vortex (g1) there is also a noticeable attached vortex (g2) at the proximal edge of the hind-wing. This additional edge-vortex induces a strong suction pressure on the ventral portion of the hind-wing and enhances thrust while reducing lift (see Fig. 5). As described above, the AoI as well as the AoA of the TOW model during the wingbeat are the same as those of the OBW model. Consequently the vortex structures produced by the TOW wing are mostly indistinguishable from those produced by the OBW wing.

### OBW VS. Other Models

**Figure 7:**
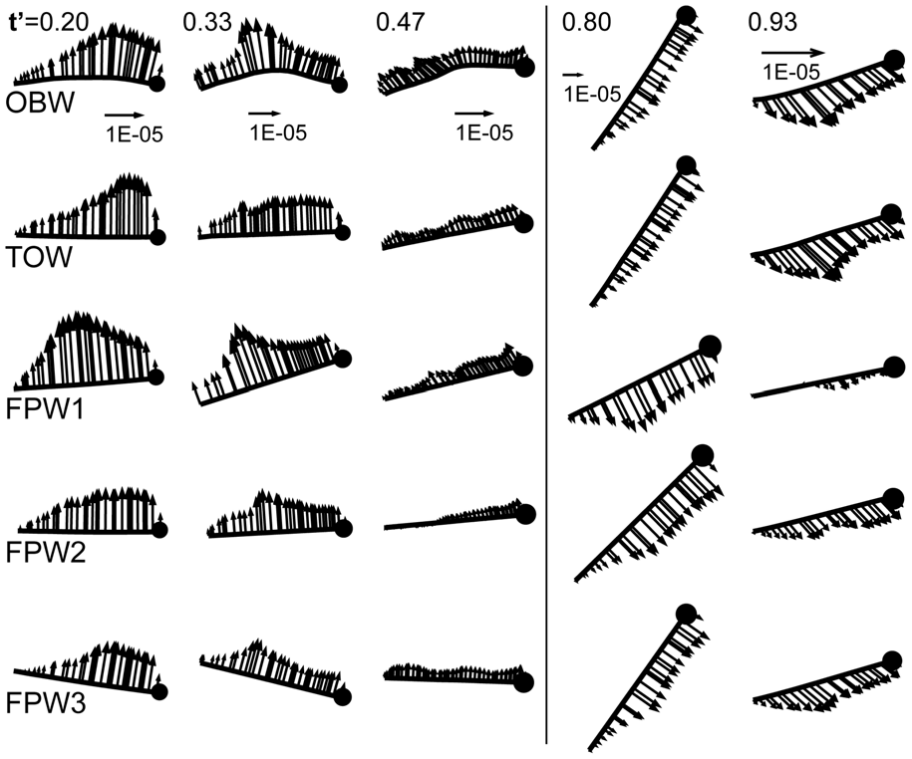
Comparison of aerodynamic traction vectors at 2/3 of the wing span during one wingbeat for different models.

Figure 7 shows the traction vectors (net aerodynamic force per unit area) on the wing at 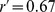. The temporal and spatial variation of this quantity enables us to relate the vortex dynamics and kinematics of each model to force production. The traction for OBW, FPW 1, FPW 3 and TOW models are compared at 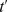 = 0.20, 0.33, 0.47, 0.80 and 0.93 in this figure. We make two general observations; first, the AoI of the FPW models is quite different from the OBW model at this 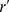. This results from the fact that by design, the FPW models match the instantaneous orientation of the wing only in some overall manner but do not match the AoI of the OBW at any particular chord location. In contrast, the TOW model matches the effective AoI of the OBW wing at this span location (as it does at all other span locations). Second, the TOW model exhibits a surface traction distribution during downstroke (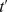 = 0.20, 0.33, 0.47) and upstroke (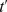 = 0.80 and 0.93) that, in totality, is the best match to the OBW among all the wing models. In particular, the TOW model exhibits high suction near the leading edge at 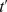 = 0.20 which is consistent with the presence of the LEV (see Fig. 6A) and also matches very closely the traction of the OBW during upstroke. The FPW 1 model produces high suction pressure (and therefore lift) during early downstroke (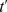 = 0.20 and 0.33) but that lift diminishes rapidly during the latter half of the downstroke, see 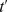 = 0.47. During early upstroke (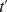 = 0.80) FPW 1 also experiences large surface traction most of which is pointed in the negative lift direction. In contrast, FPW 2 and 3 produce lower suction during the entire downstroke but during upstroke, matches the surface traction distribution of the observed butterfly wing.

Wing camber is most prominent during middle and late downstroke (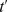 = 0.33, 0.47) and its effect is particularly evident when comparing the traction on the OBW and TOW models at 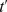 = 0.47. The presence of the positive camber in the OBW wing enables it to “catch” more air on the ventral surface and produce higher positive pressure on the ventral surface resulting in a slower drop in the lift of the OBW wing as compared to the TOW wing (see Fig. 5A).

## Summary

The effects of camber and twist have been explored by synthesizing and modeling the flow for a set of distinct wing models which match different kinematic features of an actual butterfly wing during forward flight. The twist-only wing (TOW) model has no camber but matches the time-varying AoI of the butterfly wing at every section along the span. The three flat-plate wing (FPW) models (Fig. 2) have no camber or twist but match planes formed by different sets of point-triplets on the wing surface: FPW 1 matches the motion of the forewing and hindwing as a single plate, FPW 2 matches the motion of the forewing and FPW 3 matches the motion of the leading-edge of the forewing. Comparison of AoI and AoA for the various FPW models indicates that they span a large range of possible wing kinematics around the actual observed, deforming wing of the butterfly.

The study indicates that the observed butterfly wing (OBW), which incorporates significant time-varying deformation in camber as well as twist, far outperforms all the flat-plate wings in terms of the mean forces (lift, and total force) as well as power-specific force generation (lift over power and force over power). The TOW model, which incorporates twist but no camber, comes closest to matching the performance of the OBW. This indicates that the vast majority of the benefits of wing deformation are derived from spanwise twist, despite the relatively low aspect-ratio and large camber of butterfly wings. Spanwise twist allows the proximal and distal portions of the wings to operate at different angles-of-incidence (AoI). During upstroke, the distal part of the wings translates at high AoI, which decreases local AoA; this prevents the generation of a strong LEV beneath the wings that can produce large negative vertical forces. The proximal portion of the wing operates at low AoI and avoids rapid rotation at the end of upstroke, which would generate a large negative lift peak, a positive thrust peak and thus a large power peak (see 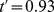 in Fig. 5). During downstroke, the wings operate at small AoI, which increases the vertical component of the aerodynamic forces and decreases the horizontal component (drag during downstroke). The chord-wise deformation (quantified by local camber) is less important than the spanwise deformation (quantified by local AoI) especially during upstroke when the local camber is almost negligible. During the latter half of the downstroke the camber “catches” air on the ventral side of the wing and by doing so, extends the width of the peak vertical force.

This study, like most other Navier-Stokes computational fluid dynamic analyses of animal flight, is limited to a single species and flight behavior due to the complexity of data acquisition, modeling, simulation and analysis. The current study is based only a single wingbeat from a single butterfly specimen. Despite the fact that the particular recording was chosen from a multitude of recordings and in our view, is a good representation of level forward flight, the generality of the findings is uncertain and a broad assessment of the effects of wing flexibility and deformation on flapping flight requires comparison of the current results to studies. In this regard, other recent work on deforming flapping wings has focused on the effects of chordwise (camber) deformation [17], [19], [28], [34]. However, our results indicate that wing twist plays a dominant role in deformation-induced enhancement of flight performance of the butterfly examined here. This may be partially due to the unique wing configuration of butterflies. Although the low-aspect ratio butterfly wings provide much scope for camber, they also increase the importance of twist because untwisted wings leave large, drag producing wing surfaces exposed to the flow. Additionally, our results and those of Young et al. [19] suggest that wing twist becomes more important in forward flight where the angles-of-incidence and attack diverge. The current study also has important implications for the design of flapping-wing micro-air vehicles (MAVs). Since the observed wing camber in butterflies (as in many other insects) does not match that adopted by a passive, flexible plate, it is difficult to replicate in an engineered system. In contrast, the observed wing-twist is generally consistent with that produced passively through flow-induced (and perhaps inertial) torques and its magnitude and phase might be controlled by appropriately tuning the torsional rigidity of the wing. This feature is relatively easy to incorporate into an engineered flapping wing and the current study indicates that significant performance benefits could be derived from such a design.
